# Glucose-6-Phosphate Dehydrogenase Deficiency Presenting as Atypical Hemolytic Uremic Syndrome: A Case Series and Literature Review

**DOI:** 10.1155/crin/1938644

**Published:** 2025-09-25

**Authors:** Ghada Almasri, Abdulkarim AlAnazi, Khawla Rahim, Hassan Faqeehi, Sawsan Albatati, Saeed Alzabali

**Affiliations:** ^1^College of Medicine, Alfaisal University, Riyadh, Saudi Arabia; ^2^Department of Pediatric Nephrology, King Fahad Medical City, Riyadh, Saudi Arabia

**Keywords:** acute kidney injury, atypical hemolytic uremic syndrome, glucose-6-phosphate dehydrogenase deficiency, hemolysis

## Abstract

Atypical hemolytic uremic syndrome (aHUS) is a severe condition marked by microangiopathic hemolytic anemia, thrombocytopenia, and acute kidney injury (AKI). It may result from complement gene mutations or be triggered by other underlying conditions. Glucose-6-phosphate dehydrogenase (G6PD) is an enzyme that protects red blood cells, and its deficiency can cause hemolytic anemia when triggered by certain factors. We report two cases of children diagnosed with G6PD deficiency who initially presented with clinical features of aHUS. In both cases, young boys developed severe AKI and hemolysis, requiring dialysis and treatment with complement inhibitors. A genetic study identified pathogenic mutations in the G6PD gene. Misdiagnosis delayed appropriate management of their underlying condition, highlighting the importance of considering G6PD deficiency in the differential diagnosis of hemolytic anemia, particularly in pediatric patients from high-risk ethnic backgrounds or those with severe hemolysis. To our knowledge, this is the first reported case series in Saudi Arabia linking G6PD deficiency to clinical presentations of aHUS.

## 1. Introduction

Atypical hemolytic uremic syndrome (aHUS) is a rare form of thrombotic microangiopathy, primarily characterized by thrombocytopenia (platelet count < 150,000/mm^3^ or a 25% reduction from baseline), microangiopathic hemolytic anemia (hemoglobin < 10 g/dL), and acute kidney injury (AKI), manifesting as proteinuria, hematuria, hypertension, and azotemia [1, 2]. aHUS can also present with progressive kidney damage or extrarenal involvement affecting other organs [1]. With an annual incidence of 1-2 cases per million, aHUS is largely caused by dysfunctional complement regulation, leading to uncontrolled complement activation on endothelial cells and platelets via the alternative pathway [3]. Most aHUS cases involve heterozygous mutations in complement components, including loss-of-function mutations in regulatory proteins such as Factor H, Factor I, MCP/CD46, and thrombomodulin, or gain-of-function mutations in C3 or Factor B [4]. aHUS can be idiopathic or triggered by factors such as upper respiratory infections, fever, pregnancy, certain drugs (e.g., quinine), or non-*E.coli* diarrheal illnesses [3]. Extrarenal manifestations occur in about 20% of cases, often affecting the central nervous system [3]. Hypertension and malaise are common nonspecific symptoms, often due to renal involvement. Clinical symptoms vary with the extent of microvascular injury, thrombosis, and ischemic damage in affected organs [2]. Initially, plasma therapy was the standard treatment for aHUS, but it was associated with high mortality and chronic kidney disease in over 50% of patients [5]. Recent studies show that C5 inhibitors, monoclonal antibodies targeting the complement component C5, have significantly improved survival rates, disease remission, kidney replacement therapy requirements, and overall quality of life [6].

Glucose-6-phosphate dehydrogenase (G6PD) deficiency, on the other hand, is a genetic disorder that affects red blood cells, making them more vulnerable to oxidative damage. This X-linked disorder is caused by mutations in the *G6PD* gene, impairing the production of the G6PD enzyme, a key component of the pentose phosphate pathway that protects red blood cells from oxidative stress [7]. Without adequate enzyme levels, red blood cells break down prematurely, leading to hemolysis [8]. G6PD deficiency is one of the most common red blood cell enzyme disorders, affecting over 400 million people worldwide [9]. It is particularly prevalent in regions with a history of malaria, such as parts of Africa, the Mediterranean, and Southeast Asia. This prevalence is due to an evolutionary advantage, as G6PD deficiency provides partial protection against malaria, as the parasite struggles to survive in affected red blood cells [10]. However, G6PD deficiency can lead to hemolytic anemia, triggered by certain drugs, foods (such as fava beans), or infections that cause oxidative stress [11]. While many individuals with G6PD deficiency remain asymptomatic, others may experience acute hemolysis episodes requiring medical attention [12]. Understanding and managing triggers is crucial to preventing complications in these patients.

Due to clinical similarities between aHUS and G6PD deficiency, particularly in cases involving hemolysis, distinguishing between them can be challenging, especially in pediatric patients. This case series highlights two children who were initially misdiagnosed with aHUS but were later found to have G6PD deficiency.

## 2. Case Presentation

### 2.1. Case 1

A previously healthy 3-year-old boy, born to consanguineous parents, presented (Day 0) with persistent vomiting (up to 10 episodes/day), yellowish discoloration of the skin, decreased urine output, generalized swelling, and reduced activity and consciousness. About a week earlier, he had experienced fever, runny nose, cough, and diarrhea.

On admission, he was hypertensive. Laboratory investigations revealed severe anemia with hemoglobin 6.0 g/dL (normal: 11.5–13.5 g/dL), thrombocytopenia with platelets around 80 × 10^9^/L (normal: 150–400 × 10^9^/L), and AKI with creatinine 257 μmol/L (normal: 27–62 μmol/L) and urea 4.25 mmol/L (normal: 1.8–6.4 mmol/L). The urine protein/creatinine ratio was markedly elevated at > 0.58 g/mmol (normal: < 0.02 g/mmol). He required an urgent packed red blood cell transfusion and was admitted to the pediatric intensive care unit with Stage 3 AKI.

By Day 1, he developed pulmonary edema, requiring intubation and mechanical ventilation. Peritoneal dialysis was initiated, and antihypertensive medications were started. Between Days 2 and 6, he received approximately five packed red blood cell transfusions for ongoing hemolysis and intravenous methylprednisolone. Urine culture grew *Candida parapsilosis*. He developed abdominal pain, and peritoneal dialysis fluid analysis revealed a white blood cell count of 0.315 × 10^9^/L (38% neutrophils), consistent with peritonitis. He was treated with ceftazidime and cefazolin. ADAMTS-13 activity and stool culture for Shiga toxin–producing *Escherichia coli* were negative, thereby ruling out thrombotic thrombocytopenic purpura (TTP) and typical HUS.

On Day 7, given the clinical suspicion of aHUS, he was started on ravulizumab (a long-acting C5 complement inhibitor), penicillin prophylaxis, and the quadrivalent meningococcal conjugate vaccine (MCV4). On Day 10, renal biopsy showed focal segmental glomerulosclerosis (FSGS), with 8 of 22 glomeruli globally sclerosed and 5 with segmental scarring. Between Days 11 and 21, his urine output and blood pressure improved, allowing cessation of dialysis, although serum creatinine remained elevated.

Although his symptoms and multiorgan involvement initially pointed toward aHUS, certain features such as ongoing severe hemolysis despite multiple transfusions, high reticulocyte count, and normal platelet count prompted consideration of alternative causes, including G6PD deficiency. He was discharged on Day 21 in stable condition on chronic kidney disease medications with outpatient nephrology follow-up. Twenty days postdischarge, G6PD testing revealed a low enzyme level of 1.8 U/g Hb (normal: 6.4–18.7 U/g Hb), confirming G6PD deficiency. Subsequently, whole exome sequencing identified pathogenic variants in the G6PD gene, and ravulizumab therapy was discontinued.

The family received detailed counseling on G6PD deficiency, including its X-linked inheritance and lifelong risk of hemolytic crises triggered by fava beans, certain drugs (e.g., sulfonamides, nitrofurantoin, dapsone, primaquine, and high-dose vitamin C), and naphthalene exposure. They were provided a written list of contraindicated substances, advised to inform healthcare providers of the diagnosis, and instructed to seek urgent care if symptoms of hemolysis such as jaundice, dark urine, pallor, or fatigue occurred. Genetic counseling was recommended. Three months after the initial presentation, the patient was clinically stable, with follow-up laboratory results summarized in Table 1.

### 2.2. Case 2

A 6-year-old male with autism spectrum disorder (ASD) presented with fever, dark red urine, jaundice, and paleness without any signs of an upper respiratory tract infection. Two weeks before, he had experienced fever and sore throat, for which he was treated with antipyretics and two doses of ceftriaxone at a private hospital. During that period, he also developed yellow vomiting, semiloose yellow stools, decreased oral intake, generalized fatigue, and increased irritability.

On presentation (Day 0), he was visibly jaundiced and mildly dehydrated, with a soft, nontender abdomen and no organomegaly. Cardiovascular and respiratory examinations were unremarkable. Vital signs included blood pressure 89/54 mmHg, temperature 37.1°C, and oxygen saturation 91% on room air. Laboratory results revealed severe anemia with hemoglobin 4.6 g/dL (normal: 11.5–13.5 g/dL), AKI with creatinine 250 μmol/L (normal: 27–62 μmol/L) and urea 27 mmol/L (normal: 1.8–6.4 mmol/L), and evidence of ongoing hemolysis. Despite four units of packed red blood cell transfusions, hemolysis persisted.

Over the next 48 h (Days 1–2), renal function worsened, with creatinine increasing to 450 μmol/L and urea to 40 mmol/L, despite aggressive hydration. The patient became oliguric. Further investigations revealed a negative direct antiglobulin (Coombs) test and negative sickle cell screening. Creatine kinase was elevated at 2871 U/L (normal: 55–170 U/L), and the peripheral blood smear showed schistocytes. Stool culture was negative for Shiga toxin–producing *Escherichia coli,* making typical HUS unlikely.

On Day 3, treatment with eculizumab was initiated (induction dose 600 mg IV × 2 doses) due to suspicion of aHUS. The patient was fully immunized prior to therapy. He underwent several sessions of hemodialysis, resulting in gradual improvement in urine output and recovery of renal function to baseline. The hemodialysis catheter was removed, and he was discharged in stable condition.

Given the combination of severe anemia (hemoglobin 4.6 g/dL), rapid transfusion requirements within a few days, persistent hemolysis despite supportive care, high reticulocyte count, and normal platelet count, which are features atypical for classic HUS, the possibility of G6PD deficiency was considered early in the course. In addition, unlike most HUS patients who present with significant hypertension, this patient's blood pressure was low-normal, further raising suspicion for an alternative diagnosis. G6PD testing confirmed deficiency with enzyme levels below normal, and genetic analysis detected a pathogenic G6PD mutation. Based on the enzyme assay results, eculizumab treatment was discontinued.

The family received extensive counseling on G6PD deficiency, including inheritance, triggers to avoid (e.g., fava beans, certain medications, and naphthalene), and the importance of informing healthcare providers. They were provided with a detailed list of contraindicated substances and instructed to seek prompt medical attention if hemolysis symptoms arise. Genetic counseling was also advised. Follow-up 2 years after the initial presentation showed that the patient remained clinically stable, with no evidence of recurrent hemolysis and preserved renal function (creatinine: 60 μmol/L, no proteinuria). There were no episodes of G6PD-related hemolysis during this period (Table 1).

## 3. Discussion

G6PD deficiency typically manifests as hemolysis triggered by infections or medications, usually without AKI or thrombocytopenia. However, AKI can occur in cases of severe hemolysis and is referred to as pigment-induced AKI [7]. In this case series, both patients showed signs resembling aHUS, including hemolysis and AKI, which initially led to the use of complement inhibitors for treatment. These cases emphasize the importance of considering G6PD deficiency, particularly in regions where it is prevalent. aHUS typically involves complement dysregulation, requiring treatment with C5 inhibitors [13]. In contrast, hemolysis in G6PD deficiency is caused by oxidative stress rather than complement activation, rendering complement inhibitors unnecessary or ineffective once the diagnosis is confirmed [7]. G6PD deficiency is highly prevalent in Saudi Arabia, with an 8.4% incidence among males, as reported by the Ministry of Health. The prevalence and genetic mutation patterns of G6PD deficiency vary across provinces, with common variants such as Mediterranean and A-, as well as rare ones such as Nara, Sibiri, and Viangchan, being identified [14]. Early detection, characterization, and understanding of the phenotypic and molecular patterns of G6PD deficiency in Saudi Arabia are essential for better management and control of the disease. Neonatal screening for G6PD deficiency, family counseling, and public awareness should be established.

In the first case, the patient's presentation of severe anemia, renal impairment, and thrombocytopenia initially raised concerns for aHUS. The introduction of ravulizumab led to clinical improvement, though the exact benefit in this context is unclear, as G6PD deficiency typically does not respond to complement inhibitors. The second case involved a patient with ASD, who presented with hemolysis and AKI, again initially suspected to have aHUS. Despite eculizumab treatment, ongoing hemolysis raised concerns for an alternative diagnosis, prompting testing for G6PD deficiency. The gradual improvement in renal function after hemodialysis and the confirmation of G6PD deficiency suggest that the patient's hemolysis was likely triggered by an oxidative stressor rather than complement dysregulation. Early identification of G6PD deficiency could have spared the patient from unnecessary complement inhibition.

These cases align with previous reports in the literature describing G6PD deficiency mimicking aHUS in pediatric populations. For instance, a case report detailed a 4-year-old boy with prior treatment for a presumed UTI, who presented with severe anemia, hematuria, AKI, and intravascular hemolysis. Initial workup ruled out Shiga toxin–associated HUS and other aHUS causes, leading to a presumptive diagnosis of primary complement-mediated aHUS. The patient was treated with plasma exchange, followed by eculizumab, which initially improved his condition. However, he experienced recurrent hemolysis with infections. Genetic testing later revealed G6PD deficiency, explaining his chronic hemolysis. Eculizumab was discontinued, and 3 years later, he remained relapse-free, though with ongoing mild hemolysis managed with folate supplementation [15].

Another report described a 2-year-old child presenting with hemolysis and thrombocytopenia in the context of an infection. Initial investigations characterized the anemia as hemolytic due to elevated LDH and hemoglobinuria, despite its nonregenerative nature. HUS was initially suspected but ruled out due to the absence of schistocytes and renal involvement. After excluding other potential causes of hemolysis, including immunological, infectious, toxic, and constitutional factors, G6PD deficiency was considered as a possible diagnosis. On the third day, methemoglobinemia (> 5%) was identified, linked to the oxidative stress of G6PD deficiency. Despite atypical features such as nonregenerative anemia and thrombocytopenia, cytological analysis of blood smears revealed hemighosts and ghosts, confirming the diagnosis and avoiding invasive bone marrow testing. Thrombocytopenia and erythroblastopenia were attributed to the viral infection, while the absence of schistocytes ruled out thrombotic microangiopathy. Molecular analysis confirmed G6PD deficiency, explaining the clinical presentation [16] (Table 2).

One limitation in both cases is that metabolism-related causes were not explored before starting anticomplement therapy, particularly cobalamin metabolism defects (e.g., cblC and cblG). While metabolic testing is not something we routinely do unless in certain situations such as infantile-onset HUS. This is important as some metabolic disorders have specific, potentially curative treatments that are different from complement inhibition.

## 4. Conclusion

This case series highlights the potential for misdiagnosis of G6PD deficiency as aHUS in pediatric patients due to overlapping clinical presentations. A detailed history, including recent infections, medication use, and food intake, combined with appropriate diagnostic tests such as a G6PD enzyme assay, is essential for accurate diagnosis and appropriate management. Early recognition and avoidance of oxidative stressors can significantly reduce morbidity in children with G6PD deficiency. Incorporating genetic testing and functional assays early in the diagnostic process can help prevent unnecessary interventions and guide appropriate management. Therefore, it is important to consider G6PD deficiency in pediatric patients presenting with hemolysis and kidney injury.

## Figures and Tables

**Table 1:** Follow-up labs.

	Case 1 (3 months from presentation)	Case 2 (2 years after discharge)
Hemoglobin (11.0–15.0 g/dL)	9.9	12.4
Hematocrit (> 35.0–< 45.0%)	31.0	37.0
MCV (> 75.0–< 95.0 fL)	67.0	90.7
MCH (> 24.0–< 30.0 pg)	21.4	30.4
RDW (> 11.0–< 15.0%)	15.0	10.8
Albumin (28.0–44.0 g/I)	37.0	39.3
Urea (2.5–6.0 mmol/L)	20.8	4.2
Creatinine (18–38 μmol/L)	115	60
Sodium (138–145 mmol/L)	131	135
Calcium (2.22–2.70 mmol/L)	2.40	2.28

**Table 2:** The clinical findings of our G6PD deficiency cases, as well as those from reported cases.

Cases	Current study		Case 3	Case 4
	Case 1	Case 2	Walsh al.	Roelens al.
Age of presentation (years)	3	6	4	2

Karyotype	46, XY	46, XY	46, XY	46, XY

Initial signs and symptoms	Fever, vomiting, jaundice, decreased urination, generalized swelling, and decreased activity and consciousness	Fever, dark red urine, jaundice, and paleness	Coryzal symptoms and macroscopic hematuria	Fever 39°C, dry cough, fatigue, and anemia

Hypertension	No	No	No	No

Hemoglobin g/dL (11.0–15.0)	9.9	4.6	4	9.9

Platelets 10^9^/L (150,000–450,000)	198,000	259,000	94,000	345,000

LDH U\L (125.00–220.00)	468.00	2790	1590	1769

Reticulocytes % (> 0.5–< 1.5%)	4.38	17.66	NA	15 G/L IRF = 5.3%

RBC fragments (negative)	Slight	NA	NA	NA

Direct Coombs test (negative)	Negative	Negative	Negative	Negative

Creatinine μmol/L (27.00–54.00)	527.00	253.00	3.3 mg/dL	31

Urea mmol/L (2.50–6.00)	11.90	27.00	NA	2.8

Albumin g/L (38.00–54.00)	23.00	39.90	NA	NA

C3 g/L (0.9–1.8)	1.21	0.914	1.22	Normal

C4 g/L (0.1–0.4)	0.49	0.233	0.24	Normal

CH50 U/mL (41.68–95.06)	> 100.00	NA	NA	Normal

Urine culture (negative)	Positive	NA	NA	NA

Blood culture (negative)	Negative	NA	NA	NA

Stool culture for STEC (negative)	Negative	Negative	Negative	NA

ADAMTS-13 activity IU/mL (0.4–1.3)	Negative	NA	113%	Normal

24-h urine protein g/day (< 0.30 g/d)	0.09	NA	NA	NA

(DBS) Glucose 6 phosphate dehydrogenase (≥ 2.2 U/g Hb)	2.8	NA	0.4 (normal: 4.5–13.5 IU/10^12^ erythrocytes)	6.5

Renal biopsy findings	Focal segmental glomerulosclerosis (FSGS)	NA	NA	NA

Gene	G6PD gene	G6PD gene	G6PD gene	G6PD gene

Variant	chrX:153,762,634 NM_001042351.1:c.563C > T(p.Ser188Phe)	NA	c.1160G > A, pR387H	c.202G > A (p.Val68Met)

Classification	Pathogenic	NA	Pathogenic	Pathogenic

Zygosity	Hemizygous	NA	Hemizygous	Hemizygous

Treatment	Ravulizumab	Hemodialysis and eculizumab	Plasma exchange and eculizumab	NA

Outcome	The condition improved after ravulizumab, with increased urine output and stable blood pressure, allowing cessation of dialysis	The condition improved after hemodialysis, leading to increased urine output and a decrease in creatinine levels to 90	Plasma exchange led to improvements in kidney function, hemoglobin, LDH, and platelet levels. After starting eculizumab, the patient contracted metapneumovirus, causing a decline in hematologic parameters while creatinine remained stable. His third dose of eculizumab resolved the episode	NA

*Note:* LDH: lactate dehydrogenase.

## Data Availability

Data sharing is not applicable to this article as no datasets were generated or analyzed during the current study.
